# Dyspnea in an Otherwise Healthy 18-year-old: The Importance of Point-of-care Ultrasonography

**DOI:** 10.5811/cpcem.2018.9.39218

**Published:** 2019-07-01

**Authors:** Emily C. Cleveland Manchanda, Sigmund J. Kharasch, Andrew S. Liteplo

**Affiliations:** Massachusetts General Hospital, Department of Emergency Medicine, Boston, Massachusetts

## Abstract

A healthy 18-year-old male presented to the emergency department with chest pain, palpitations, and dyspnea. His exam was unremarkable; however, point-of-care ultrasound (POCUS) revealed right ventricular strain with a D-sign and enlarged right ventricle. He subsequently reported a history of factor V Leiden. His D-dimer was markedly elevated, and a computed tomography angiogram of the chest demonstrated submassive pulmonary embolism (PE). He was taken to the catheterization lab for directed thrombolysis and was discharged in good condition two days later. Factor V Leiden is the most common genetic cause of venous thromboembolism. POCUS can facilitate rapid diagnosis and risk stratification of patients with acute PE.

## INTRODUCTION

Within the field of emergency medicine, point-of-care ultrasound (POCUS) has become a widely accepted tool in the emergency physician’s (EP) arsenal for both diagnosing and exonerating pathology. Nevertheless, consulting services are often skeptical of the EP’s ultrasound findings, and at our institution frequently request confirmatory, comprehensive ultrasonography before proceeding with invasive procedures or other interventions. This case highlights the utility of expedited POCUS in the rapid diagnosis and subsequent treatment of this life-threatening condition.

## CASE REPORT

An 18-year-old male who reported no past medical history presented to the pediatric emergency department (ED) complaining of mild substernal chest pain, intermittent palpitations, and dyspnea with minimal exertion over the prior week. He endorsed decreased oral intake of fluids and solids over the prior week and occasional non-productive cough. He endorsed some caffeine intake (1–2 caffeinated sodas per day), but no recent increase in caffeine consumption. He denied any fevers, drug use including cocaine, and reported no significant familial history of hyperthyroidism, cardiac disease, or sudden death. There was no history of recent upper respiratory infections, muscle pain, back pain, joint stiffness, nausea, vomiting, diarrhea, rash, dizziness, syncope, leg swelling, or history of trauma. There were no known ill contacts.

Initial exam revealed a very well-appearing young man in no distress, speaking in full sentences, and sitting on the stretcher playing with his 9-month-old child. Initial vital signs included a temperature of 96.7 degrees Fahrenheit, pulse of 116 beats per minute (bpm), blood pressure of 126/81 millimeters of mercury (mmHg), respiratory rate of 18 breaths per minute, and an oxygen saturation of 95% on room air. There were no murmurs on cardiac exam. His pulmonary exam was normal with no wheezes or accessory muscle use. The abdomen was soft with no tenderness, and his extremity and neurologic exams were unremarkable. No lower extremity edema, calf cords, or leg tenderness was noted.

**Image 1:**
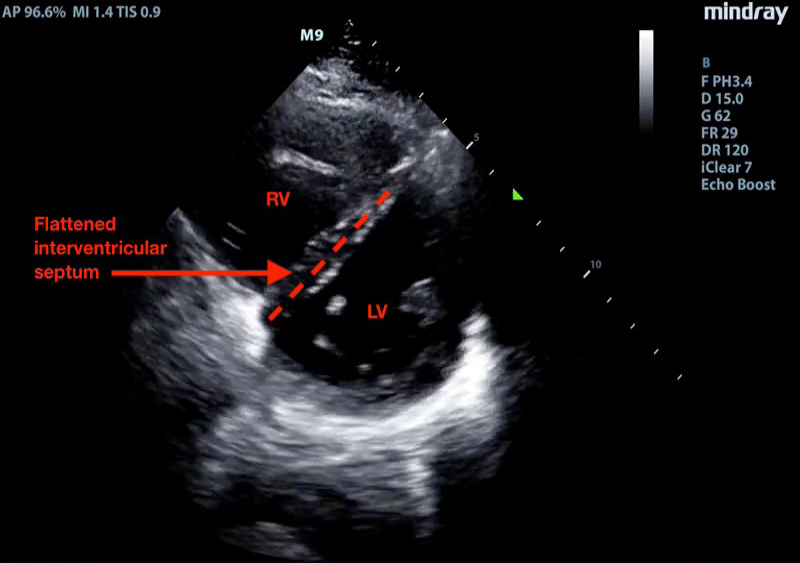
Parasternal short view of the heart, demonstrating bowing of the interventricular septum away from the right ventricle (RV) into the left ventricle (LV) (the “D-sign”).

**Image 2:**
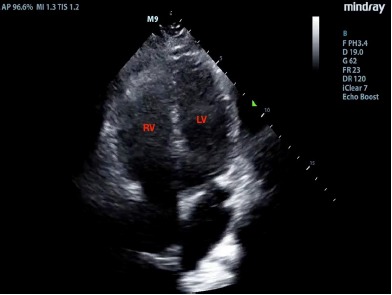
Apical four-chamber view of the heart, demonstrating an enlarged right ventricle (RV), greater in size than the left (LV).

The patient had an intravenous line placed, and initial labs included a complete blood count and electrolytes, which were normal. He was given one liter of normal saline and his heart rate improved to 92 bpm. A point-of-care cardiac ultrasound was performed. He had a normal ejection fraction on the parasternal long view with no pericardial effusion. However, the parasternal short-axis view demonstrated a “D-sign,” wherein bowing of the interventricular septum into the left ventricle (LV) caused it to take on the shape of a capital letter D, indicative of right ventricular (RV) strain (Image 1 and Video 1). The apical four-chamber axis view demonstrated an enlarged RV, although the team did not observe akinesis of the mid-free wall of the RV (“McConnell’s sign”) (Image 2 and Video 2). A D-dimer was sent, as the identification of RV strain on POCUS increased the team’s suspicion for pulmonary embolism (PE). Ultrasound of the lower extremity veins identified a non-compressible right popliteal vein, indicative of an occlusive thrombus.

On further questioning regarding family history, the patient called his mother and subsequently reported that he carried a diagnosis of factor V Leiden mutation, which had been identified after his mother developed an unprovoked PE several years prior. He also recalled twisting his right ankle one week prior to presentation, although he had no significant swelling or difficulty walking thereafter.

**Image 3:**
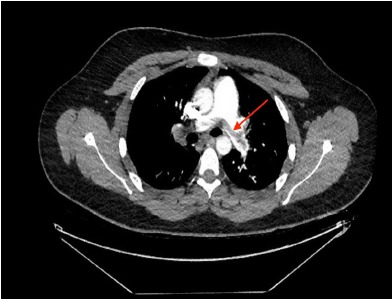
Axial view of the chest on computed tomography for pulmonary angiogram, demonstrating a saddle pulmonary embolism (arrow), with near-occlusive clot in the right pulmonary artery.

The patient’s D-dimer subsequently came back at greater than 6,000, with a N-terminal pro b-type natriuretic peptide (NT-proBNP) elevated at 1096. Troponin was negative. Computed tomography (CT) angiography of the chest identified a saddle PE, with near-occlusive clot in the right pulmonary artery, signs of right heart strain, and pulmonary arterial enlargement (Image 3). The patient was taken to the cardiac catheterization suite for ultrasound-assisted, catheter-directed, low-dose thrombolysis using the EkoSonic Endovascular System (EKOS catheters). He was admitted to the medical intensive care unit thereafter and recovered without complications. He was discharged two days later on rivaroxaban, with the plan for lifelong anticoagulation.

**CPC-EM Capsule**

What do we already know about this clinical entity?

Pulmonary embolism (PE) can cause significant morbidity and mortality. Point-of-care ultrasonography (POCUS) can rapidly identify right heart strain, which may predict clinical outcomes following PE.

What makes this presentation of disease reportable?

Despite having a submassive PE with a large clot burden, the patient was only mildly symptomatic. Without POCUS, his PE could have been missed, leading to long-term cardiopulmonary sequelae.

What is the major learning point?

Use POCUS to evaluate patients with cardiopulmonary symptoms in the emergency department. Signs of right heart strain may lead to more rapid diagnosis and treatment of potentially fatal PEs.

How might this improve emergency medicine practice?

Early use of POCUS to evaluate patients with cardiopulmonary symptoms in the emergency department can critically alter their care.

## DISCUSSION

POCUS is a useful tool for expediting the diagnosis of venous thromboembolism (VTE) in the ED.^1^ Emergency physicians may obtain reasonable competency in POCUS diagnosis with even limited training.^2^ The parasternal long-axis view is most useful for observing global LV function, as well as for identifying a pericardial effusion. The parasternal short-axis view is useful for identifying wall motion abnormalities in the LV, as well as for comparing the relative pressures between the LV and RV. When right-sided pressures are elevated, as seen in this case, the interventricular septum bows toward the left during diastole, distorting the LV from its usual rounded appearance into a D-shape. In the apical four-chamber axis view, the RV diameter is usually no more than 70% that of the LV measured at the level of the tips of the valves at end-diastole. An RV that is *greater* in size than the LV in the apical four view is pathologic and indicates elevated RV pressures.^3^ McConnell’s sign – akinesis of the mid-free wall of the RV with normal motion at the apex, best seen in the apical four-chamber axis view – was initially described to have a relatively low sensitivity (77%) but high specificity (94%) for acute PE.^4^ A subsequent study, however, showed that acute RV infarction has a similar incidence of McConnell’s sign as PE, suggesting that perhaps it is more indicative of an acute process rather than RV failure due to PE in particular.^5^

Even in the era of CT angiography, ultrasound continues to play an important role in risk stratification.^6^ In a prospective study of patients with acute PE, Grifoni et al. demonstrated that among initially normotensive patients, RV dysfunction on ultrasound was associated with a 10% rate of subsequent PE-related shock and 5% in-hospital mortality, while patients without RV dysfunction had benign inpatient courses.^7^ Patients with intermediate-risk PEs, defined as PE with evidence of RV dysfunction and/or myocardial injury without hypotension or shock,^8^ have a mortality rate of 3%, nearly the same as patients who present with acute myocardial infarction.^9^ Retrospective studies have shown that 80% of patients with saddle PEs demonstrate RV dysfunction, and have a mortality of between 5–6%.^6^,^10^,^11^ These patients may be candidates for catheter-directed thrombolysis in addition to systemic anticoagulation.^12^ Some data suggests that patients such as the one in this case have improved outcomes after ultrasound-assisted, catheter-directed thrombolysis compared to systemic anticoagulation alone, without an increased rate of bleeding complications.^13^

Factor V Leiden is the most common genetic risk factor for VTE.^14^ Approximately 3–8% of the general United States and European populations are heterozygous carriers of the modified allele. It is characterized by poor response to activated protein C and leads to increased risk of deep vein thrombosis and PE. Individuals with factor V Leiden mutations are at a 50-fold increased risk for VTE after a minor lower extremity injury compared with individuals without this mutation,^15^ and heterozygous carriers have been shown to have a lifetime VTE risk of 10% in population studies.^16^

## CONCLUSION

This case highlights the power of POCUS in the early evaluation of patients with cardiopulmonary complaints. The recognition of right heart strain early in this patient’s course led the clinical team to obtain an urgent CT angiogram, which facilitated the timely diagnosis for his submassive PE. Despite his large clot burden, the patient was only mildly symptomatic. Without POCUS, his PE could have been missed as his heart rate normalized and some of his symptoms improved following administration of intravenous fluids. In this case, the EP’s ultrasound, in conjunction with the clot burden seen on CT, were conclusive evidence of significant RV dysfunction, and prompted the cardiac surgery team to take the patient directly to the catheterization laboratory for thrombolysis without a comprehensive transthoracic echocardiogram. His expedited diagnosis in the ED led to rapid intervention and treatment by the appropriate specialist team and may have avoided long-term cardiopulmonary sequelae.

## Supplementary Information

Video 1. Ultrasound video clip of parasternal short view of the heart, demonstrating bowing of the interventricular septum away from the right ventricle (RV) into the left ventricle (LV) (the “D-sign”).

Video 2. Ultrasound video clip of apical four-chamber view of the heart, demonstrating an enlarged right ventricle (RV), greater in size than the left (LV).
